# Factors Associated with Persistent Lower Respiratory Symptoms or Asthma among Residents Exposed to a Sulphur Stockpile Fire Incident

**DOI:** 10.3390/ijerph16030438

**Published:** 2019-02-02

**Authors:** Roslynn Baatjies, Shahieda Adams, Eugene Cairncross, Faieza Omar, Mohamed F. Jeebhay

**Affiliations:** 1 Department of Environmental and Occupational Studies, Faculty of Applied Sciences, Cape Peninsula University of Technology (CPUT), Cape Town 7535, South Africa; Roslynn.Baatjies@uct.ac.za; 2 Occupational Medicine Division and Centre for Environmental and Occupational Health Research, School of Public Health and Family Medicine, University of Cape Town, Observatory 7925, South Africa; Shahieda.Adams@uct.ac.za; 3 Emeritus Professor, Department of Chemical Engineering, Cape Peninsula University of Technology (CPUT), Cape Town 7535, South Africa; cairncrosse@gmail.com

**Keywords:** persistent lower respiratory symptoms, asthma, exposure, sulphur dioxide

## Abstract

*Introduction:* Residents of Macassar, South Africa, were exposed to sulphur dioxide vapours (SO_2_) caused by an ignited sulphur stockpile, which produced peak hourly SO_2_ levels of 20–200 ppm. The aim of this study was to assess the risk factors associated with persistent lower respiratory symptoms (LRS) or asthma six years after acute exposure to high SO_2_ levels. *Methods:* A case-control study of residents that presented for a health evaluation six years after the incident was conducted. Survey instruments included a questionnaire, clinical examination and medical record review by an expert panel. A “case” was defined as a resident with persistent LRS/asthma. The Industrial Source Complex Short Term Model (ISCST 3) was used to predict time-averaged hourly SO_2_ levels. *Results:* A previous history of pulmonary tuberculosis (PTB) was associated with persistent LRS/asthma (OR_udj_: 3.49, CI: 1.46–8.35). Cases were more likely to report chest tightness (OR_udj_: 9.93; CI: 5.15–19.11) at the time of the incident. Peak exposure at hour 15 was associated with persistent LRS/asthma (OR_adj_: 1.04; CI: 1.01–1.07). *Conclusion:* LRS/asthma persisted in some individuals six years after acute SO_2_ exposure_._ Aside from peak exposures, initial chest tightness and a previous history of PTB were the strong predictors of persistent LRS/asthma.

## 1. Introduction

Exposure to sulphur dioxide (SO_2_) has been associated with increased risk of respiratory and total mortality [1], asthma diagnosis [2], exacerbation of pre-existing respiratory disease [3] and respiratory symptoms such as wheezing and shortness of breath [4]. Various epidemiological studies have demonstrated changes in respiratory symptoms and pulmonary function in individuals with asthma following exposure to SO_2_ for a duration as short as 10 minutes [5,6,7,8]. 

A limited number of studies have documented long term respiratory morbidity among individuals that have been acutely exposed to high levels of SO_2_ following an incident. The most commonly reported adverse respiratory health effects include bronchial hyper-responsiveness (BHR), acute irritant induced asthma (IIA) (commonly referred to as reactive airways dysfunction syndrome—RADS) and chronic obstructive pulmonary disease (COPD) [9,10,11]. Persistence of these adverse respiratory health effects in subjects exposed to SO_2_ have been reported between 3 months to 14 years after the initial acute episode [12,13].

The increased susceptibility of certain individuals to irritants such as SO_2_ is an important consideration in evaluating the long term adverse respiratory health effects following acute environmental exposure. Demeter et al. [12] reported that asthmatics may have aggravation of their symptoms and BHR nine years after excessive exposure. Furthermore, asymptomatic adults with BHR also appeared to be more susceptible to SO_2._ In industrial settings with repeated peak exposures to SO_2_ have also been associated with an increased risk of incident asthma (Hazard ratio = 4.00) [14].

More recently, a study investigated the long-term health effects of volcanic gas. A major feature of the Mount Oyama eruption was the high emission of large amounts of SO_2_, which reached 80,000 tons/day during the peak period (November 2000) and continued for a long time thereafter. Individuals exposed to short-term high dose peak exposures of SO_2_ were more likely to have irritative respiratory symptoms 6 years after the exposure but did not demonstrate any deterioration in lung function [15]. A 10-year observational study conducted in Portugal also reported a positive association between chronic exposure to volcanic gases containing SO_2_ and the incidence of chronic bronchitis recorded at local health centres serving the exposed population [11].

This current study investigated the respiratory morbidity among residents of a neighbourhood community following an incident involving a sulphur stockpile fire at a chemical manufacturing company. At the time of the disaster, the population of the nearby Macassar township was estimated to be at least 30,000 residents. It is estimated that almost half of the sulphur stockpiled on the premises (7250 tons) was consumed by the fire resulting in 14,500 tons of SO_2_, the primary combustion product, being released into the environment. [16]. Initial reports indicated that the fire raged on for about 21 h, with varying levels of intensity, producing SO_2_ concentrations ranging between 3–55 ppm, with the highest exposures (>100 ppm) being experienced in the first 7 h due to the strong prevailing north-westerly wind [16]. Over 3000 residents had to be evacuated at the time. It is reported that a large proportion (95%) of these residents experienced various acute symptoms attributable to the fire. Upper and lower respiratory symptoms (LRS) were the most common symptoms reported within the first week after the fire [16].

The aim of this study was to determine the risk factors associated with the development of persistent LRS or asthma among residents six years after acute environmental exposure to SO_2_.

## 2. Materials and Methods

### 2.1. Study Design

A case-control study was conducted over a period of 24 months. Cases and controls selected from all the affected residents underwent a rigorous 2-step medical evaluation process, which entailed administration of a medical screening questionnaire and a focussed respiratory clinical examination performed by an occupational health nurse (step 1). This was followed by a review of the collected information together with other medical records provided by their doctor by a medical reference panel (MRP) comprising occupational medicine specialists (step 2). On the basis of this process, cases and controls were selected from the study population. All cases so identified were referred to a pulmonologist to confirm the presumptive diagnosis made by the MRP. This was based on the presence and nature of respiratory symptoms (including any reports of pre-existing respiratory disease) and their relationship to the incident. Due to efficiency considerations, cost and time, controls were not evaluated further by the pulmonologist since the MRP’s opinion was considered to be sufficient.

### 2.2. Study Population

The study population consisted of all 4000 residents who suffered acute health effects (mainly upper and lower respiratory symptoms) due to acute exposure to SO_2_ vapours at the time of the fire incident. Residents also presented with other health complaints (psychological, skin and reproductive problems), but these were not considered further for the purposes of this current study. Both cases and controls were selected in accordance with predetermined criteria. All cases identified were chosen and compared to twice the number of controls in the statistical analysis.

#### 2.2.1. Definition of Cases

All cases fulfilled the following entrance criteria for being classified as a case:

- Macassar resident at the time of the fire incident
- Eighteen years or older at the time of the fire incident
- Underwent a medical evaluation at the Macassar disaster project clinic
- Free of persistent LRS, asthma and other chronic respiratory illness such as pneumonia or chronic obstructive pulmonary disease at the time of the disaster
- Absence of pulmonary TB (PTB) at least one year prior to the disaster
- Reported persistent respiratory symptoms/asthma at year 1 (Yr_1_) and year 6 (Yr_6_) after the incident which in the opinion of the medical panel (MRP) was probably related to inhaling SO_2_ vapours at the time of the fire incident

#### 2.2.2. Definition of Controls

All controls fulfilled the first 5 criteria but were free of persistent LRS or asthma following the incident.

#### 2.2.3. Definition of Persistent Lower Respiratory Symptoms or Asthma Attributable to the Fire

Persistent LRS included wheezing, a tight chest and/or a diagnosis of asthma (irritant induced asthma, asthma aggravation) attributable to the fire, and was present at Yr_1_ and Yr_6_ following the incident.

### 2.3. Data Collection

#### 2.3.1. Health Outcome Assessment

*Questionnaire*: An occupational health nurse conducted structured face to face interviews. Data obtained from this interview included demographics, exposure history and SO_2_-related symptoms experienced after the incident.

*Clinical examination:* To ensure uniformity in assessment, the same occupational health nurse responsible for the interviews performed all the examinations. The clinical examination focussed on the respiratory system.

*Discussion of medical dossier by the MRP:* A panel of at least 3 experienced occupational medicine specialists reviewed the information obtained from the questionnaire, medical records provided by the individual’s personal doctor and the clinical examination to assess the presence of persistent LRS, their relationship to the incident and the need for further investigations. 

*Specialist pulmonological assessment:* Individuals with persistent LRS were referred to the same pulmonologist to obtain an independent detailed medical history, conduct a clinical examination and other investigations (chest radiographs and lung function testing). The pulmonologist provided a final diagnosis for each individual after the clinical evaluation. Spirometry was performed according to American Thoracic Society (ATS) guidelines, and the post-bronchodilator European Community Coal and Steel workers (ECCS) prediction equations were used to compute the lung function reference values. [17,18]. This information was used to further define the nature and severity of airway obstruction present in the cases.

#### 2.3.2. Environmental Exposure Assessment

*Sulphur dioxide exposure model:* A well-validated steady-state Gaussian plume model, the Industrial Source Complex Short Term Model (ISCST3) [19] was used to predict hourly average peak and 24-hour average SO_2_ concentrations at specified receptor locations [16,20]. Parameters necessary to perform the modeling simulations were obtained from an exposure estimate model using actual exposure levels taken shortly after the disaster [16]. The parameters described the emission rate, temperature, location and extent (geometry) of the sulphur fire. Release rates were determined as a function of time based on the sequence of the fire. Since emissions occurred over an extended spatial area, 16 release points were selected to represent the source’s area and shape. Meteorological data of the location were included in the modeling simulation. 

*Individual-specific exposures:* SO_2_ concentrations were obtained for 441 receptor locations over a 4 × 2 km grid for every hour that the fire was ablaze. Exposures for each individual were taken as the average hourly concentration of the receptor nearest to the residence of the reported location of an individual at the time of the fire. In addition, a peak exposure (highest hourly exposure), total hours (time present at the location) and cumulative exposure (concentration x exposure duration) estimates were calculated for each individual.

### 2.4. Statistical Analyses

STATA version 14 (StataCorp, College Station, TX, USA) statistical software was utilised for the statistical analysis. Descriptive statistics were used, and odds ratios were calculated to compare cases and controls across the predictor variables of interest. Generalised linear models were used for multivariate logistic analyses. All models were formulated using the saturated model approach when adjusting for potential confounders so as to enable comparisons across various exposure metrics. For the statistical analysis of SO_2_ exposure concentrations, the data followed a log-normal distribution. The natural logarithm of the calculated SO_2_ exposure was therefore used as the independent variable.

## 3. Results

The demographic characteristics of the cases and controls are outlined in Table 1. A total of 76 cases and 180 controls were selected. The cases and controls were comparable with respect to age, gender and smoking. A larger proportion of the cases (17%) compared to the controls (10%) reported a previous history of PTB more than one year prior to the incident. 

Specialist pulmonological evaluation of the cases at Yr_6_ revealed that at least half of the cases with persistent LRS were diagnosed as new-onset acute irritant induced asthma (IIA/RADS) (64%) or reactive upper airway dysfunction syndrome (RUDS) (46%), with 34% having both co-conditions existing. A lower, but sizeable proportion were diagnosed with asthma aggravation (32%), and a very small proportion (4%) had COPD. The prevalence of rhinitis aggravation was only (5%).

While a large proportion (63%) of the cases had reduced lung function (FEV_1_ <80% predicted), 24% of the cases had evidence of airway obstruction on spirometry (FEV_1_/FVC <70%). The latter prevalence was very similar to the proportion (22%) of cases who only had IIA (RADS). Over a third of the cases (37%) demonstrated evidence of significant bronchial reversibility (Table 2). Among those with impaired lung function, most (42/48 = 88%) had mild impairment.

**Figure 1:**
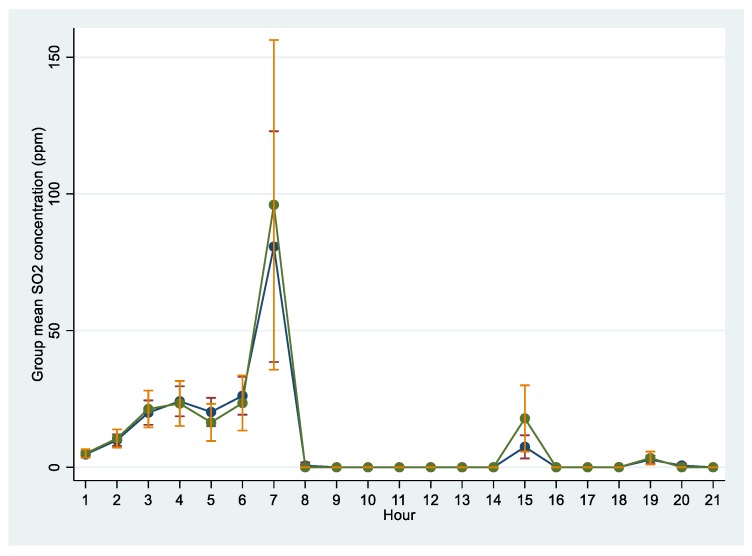
Estimated mean hourly exposures to sulphur dioxide during the sulphur stockpile fire incident according to the Industrial Source Complex Short Term Model (ISCSTM). Legend: green dotted line = cases, blue dotted line = controls.

A comparison of the summary measures of the exposure metrics for cases and controls is outlined in Table 3. The mean total hours of exposure were similar for both cases and controls. Overall, the cases experienced higher peak SO_2_ exposures and cumulative exposures than controls, but the difference was not statistically significant. During the 21-hour exposure period, two peaks of hourly SO_2_ exposures were observed (Figure 1). The first peak (hr_7_) occurred within the first eight hours of the fire prior to the commencement of the evacuation process and a second relatively smaller peak occurred at hr_15_. Although cases had higher mean peak exposures than controls at hr_7_ and hr_15_, only the latter peak was statistically significant (*p* = 0.04) between the two groups.

In the unadjusted logistic regression analysis none of the host-associated factors (age, sex, smoking status) were significantly associated with persistent LRS/asthma attributable to the incident (Table 4). Residents with persistent LRS/asthma were more likely to report a tight chest (OR: 9.93, CI: 5.15–19.11) than shortness of breath (OR: 1.97, CI: 0.95–4.09, *p* = 0.07) at the time of the incident. Interestingly, residents who reported headaches were less likely to develop persistent LRS/asthma (OR: 0.27, CI: 0.08–0.92). Furthermore, those with persistent LRS/asthma were also more likely to have a previous medical history of PTB (more than one year prior to the fire incident) (OR: 3.49, CI: 1.46–8.35).

The logistic regression analysis (unadjusted and adjusted models) for the various exposure metrics are outlined in Table 5**.** None of the exposure metrics were significantly associated with persistent LRS/asthma attributable to the fire, although the peak levels demonstrated more consistent positive associations. The peak SO_2_ exposure concentration at hr_15_ (but not hr_7_) in the multiple regression model was significantly (*p* = 0.022) associated with increased odds of having persistent LRS/asthma (OR: 1.04, CI: 1.00–1.07)

## 4. Discussion

Exposures characterised by inhalation of chemicals and particulate matter (dusts and fibers), during occupational and environmental disasters have been associated with various respiratory disorders [21]. In this study, the environmental risk factors associated with persistent LRS or asthma in residents were assessed six years after being acutely exposed to high SO_2_ vapours emanating from a sulphur stockpile fire. Despite the well-known irritant effects of SO_2_, limited information is available about the long-term health effects of acute high levels of exposure to SO_2_. The study found that Macassar residents with persistent LRS/asthma were more likely to have presented with acute asthma-related symptoms (tight chest and shortness of breath) at the time of the fire. Furthermore, exposure to SO_2_ peaks during the fire, at hr_15_ and possibly hr_7_, might have contributed to the development of persistent LRS/asthma.

Persistence of LRS has been reported in a few studies following acute and/or accidental exposure to respiratory irritants. Studies following the World Trade Center (WTC) incident, showed that rescue/recovery workers, local area workers and residents experienced a high prevalence of persistent LRS within the first 5 years of the incident [22,23,24,25]. Further follow-up studies demonstrated stabilisation of the elevated prevalence of dyspnoea and wheeze among firefighters 5–9 years later [26]. Wisnivesky et al. [27] reported an asthma prevalence of 18.1% in other rescue/recovery workers after 9 years. More recent studies demonstrated that LRS (cough, wheeze and dyspnoea) among WTC health registrants enrolees persisted for at least 10 years [28]. Furthermore, a study that investigated the long-term impact of high environmental exposures to SO_2_ present in volcanic gas, reported that irritative symptoms persisted 6 years following exposure but were not accompanied with a deterioration in the lung function of subjects [15].

Epidemiological surveys that have investigated irritant induced asthma due to single, high-level

exposures have been relatively few [29]. Retrospectively reported acute inhalation was associated with new-onset asthma in the longitudinal European Community Respiratory Health Survey [30]. Other studies have also shown that asthmatics more often have a history of single high exposure to irritant cleaning products than healthy controls [31]. An increased asthma risk in men with a history of accidental peak exposure to irritants has also been reported [32]. In a longitudinal study of rescue and recovery workers that experienced intense dust cloud exposure during the World Trade Centre incident, an increased risk of new-onset asthma was demonstrated over the 5–6 year follow up period, and more especially within the first few months after the exposure event [33].

In this study, individuals with persistent LRS six years after the incident (e.g. cases, *n* = 76) were mainly diagnosed with new-onset reactive airway dysfunction syndrome (IIA/RADS) (64%) and asthma aggravation (32%). These symptoms were considered to be directly related to the fire incident by the expert medical panel. Furthermore, smoking status was not significantly associated with having persistent LRS/asthma, strengthening the presumption that the condition was attributable to the fire. While reactive upper airway dysfunction syndrome (RUDS) was present in 46% of cases, 34% had co-existing irritant-induced RUDS and IIA (RADS) diagnoses. Demeter et al. have shown that chronic rhino-sinusitis symptoms requiring treatment may parallel the course of asthma in individuals with IIA (RADS) [12]. Overall, the findings in this current study are consistent with previous reports of an association between upper and lower airways symptoms after accidental exposure to respiratory irritants [23,34].

In this study a considerable proportion (37%) of cases had clinically significant bronchial reversibility. These findings are consistent with those reported by Piirilä et al., which demonstrated that reversible airway obstruction was still present after four years, while an altered bronchial response persisted 13 years after the initial SO_2_ exposure [13]. The proportion of cases with reduced lung function (22%) in this study was lower than that reported by Brooks et al., which showed that 40% of subjects with IIA (RADS) had chronic obstructive pulmonary disease (FEV_1_/FVC <70%) at follow up examination (mean = 35.8 months) [35]. Another study of pulp mill workers who experienced shortness of breath more than one month after a chlorine gassing incident, demonstrated significant BHR (57%) and airway obstruction (31%), after 18–24 months [36]. Malo et al. reported mixed findings in their longitudinal assessment of 35 patients with acute-onset IIA assessed after a mean interval of 14 years, with more individuals with BHR persisting (75%) than improving (39%) in this group. However, airway obstruction did not improve significantly at follow-up [37]. 

The current study also investigated the association between certain host factors and persistent LRS/asthma. While, none of the demographic characteristics were significantly associated with persistent LRS/asthma, subjects with persistent LRS/asthma were more likely to report chest tightness and shortness of breath at the time of the fire incident. This is consistent with previous studies in which LRS occurring immediately after an exposure incident was a significant factor in determining long term respiratory morbidity due to SO_2_ exposure [12,13]. The findings of this study support the recommendation of instituting more intensive and regular monitoring in those individuals who present with chest tightness at the time of acute exposure, given the increased risk of subsequently developing persistent LRS/asthma. The findings of this study suggest that premorbid respiratory health status, and more particularly having pulmonary TB (more than one year prior to the fire), could be an important factor predicting chronicity of asthma-related symptoms associated with acute SO_2_ exposure. Subjects with persistent LRS/asthma attributable to the fire were more likely to have a medical history of PTB. These findings mirror those of the national demographic health survey for South Africa at the time, which demonstrated that a history of PTB was an independent predictor of both recent wheeze (OR: 3.4; 95% CI 2.5–4.7) and asthma diagnosis (OR: 2.2; 95% CI 1.5–3.2) [38]. Airway obstruction has been a well-documented long-term sequela of PTB in other community-based studies in the country showing an increased risk (OR = 2.6–8.9) [39,40]. The strong association between previous PTB and the development of persistent LRS, asthma or chronic bronchitis has been shown in both occupational and community-based studies [41]. It has been suggested that this association may be related to post-PTB structural damage of the airways. Furthermore, immunomodulatory effects due to macrophage dysregulation leading to airway remodelling, may also cause chronic airflow obstruction [42]. The association of past PTB with asthma diagnosis is less clear and it is conceivable that this could be due to post-tuberculous obstructive lung disease being misdiagnosed as asthma [22]. However, this was considered to be unlikely in this study since all cases were evaluated by a pulmonologist using the appropriate clinical tests, including spirometry.

The constraints imposed on exposure characterisation approaches during a disaster or unanticipated incident, have resulted in most studies lacking precise data on exposure dose to potential irritants, making it difficult to study exposure response relationships for IIA and persistent respiratory function abnormalities [31]. In this study, various environmental exposure metrics were developed to study these associations. However, most of these exposure metrics were not significantly associated with the outcome of interest, except for peak SO_2_ exposure levels at hr_15_, which was associated with an increased risk of having persistent LRS/asthma attributable to the fire. Furthermore, peak exposure categories of 2–20ppm and >20ppm were also positively associated with persistent LRS/asthma in an incremental manner, although not statistically significant (*p* > 0.05). Friedman et al. were able to demonstrate significant exposure-response relationships for persistent LRS with increasing exposure scales [28], while Hall et al., also demonstrated an almost two-fold increase in obstructive airway disease in firefighters 7 years post-incident between high and low exposure groups [43]. Among workers engaged in WTC rescue and cleaning operations, the main risk factors for respiratory disease included being present on the site during the first 48 h and the duration of rescue and cleaning operations [44].

Intensity of exposure is therefore an important predictor and several reports in the literature suggest that exposure to SO_2_ > 20 ppm is associated with chronic respiratory symptoms [45,46]. The findings from the Miyakejima Island study also demonstrated that exposure to short-term high (peak) concentrations, rather than average concentrations, contributed to symptoms of respiratory irritation. Furthermore, a significantly higher prevalence of nasal irritation has been observed in specific areas due to higher maximum 5-minute concentrations in these places at that time [15]. Ishigami et al. also demonstrated that hourly average SO_2_ concentrations >10 ppb were associated with increased rates of throat irritation and cough in healthy volunteers who stayed on Miyakejima Island up to 15 days following a volcanic eruption [47].

This study provided a rare opportunity to study persistent LRS/asthma following a fire disaster associated with high levels of exposure to SO_2_ in a relatively large sample of affected population. The use of standardised medical evaluation assessments in the screening phase, confirmation of symptoms with more objective clinical tests by a pulmonologist and the availability of detailed medical records on all cases during the follow up period enabled a comprehensive evaluation of all cases. The exposure characterisation approach was novel since it included a quantitative estimate of SO_2_ exposure levels that was assigned to each study subject. The overall accuracy of modelled exposure estimates was validated against limited available ambient data. The 1-h peak SO_2_ concentrations reached 1.1 and 1.2 ppm at two monitoring stations 33 km and 38 km north-west of the fire, which is reasonably comparable to the modelled hourly isopleths of 1 and 3.5 ppm at these sites [16]. While the hour to hour uncertainty in exposure levels is acknowledged, all estimates of population level exposure-response relationships included similar levels of uncertainty and were unlikely to bias the results in a differential manner.

A limitation of this study was the inability to demonstrate consistent statistically significant exposure-response relationships across the various exposure metrics (subject location, duration of exposure, cumulative exposure) due to possible exposure misclassification since SO_2_ levels varied considerably, both temporally and spatially over the 21-hr period. It is possible that different human behaviours were at play, and that most residents remained indoors in shut houses, partly sheltered from the SO_2_ plume, while others had to be evacuated before the plume passed across their area. Batterman et al. have therefore suggested that a better understanding of exposures and health effects requires additional information on demographics (population density), housing characteristics (air exchange rate and locations) and activity patterns (location, time outdoors, etc.) in order to generate more robust exposure indicators [48].

With regard to the health outcome endpoints, potential biases need to be considered. It is possible that “over-reporting” of symptoms may be expected in this study population since the disaster project clinic was primarily set up to undertake a medico-legal process to establish eligibility of individuals for compensation pay-outs. However, this would not have been operating differentially since the entire study population would have been motivated to over-report. It is possible that ‘recall bias” may have occurred since subjects had to rely on their memory of an incident six years ago (e.g. symptoms experienced, or time spent in their home at the time of the fire). However, standardisation of the medical evaluation process enabled all cases and controls to undergo the same screening process and ensured that they were evaluated by the same occupational health nurse and medical reference panel.

## 5. Conclusions

In conclusion, this study demonstrated that premorbid history of PTB as well as acute symptoms of chest tightness and shortness of breath are important factors predicting chronicity of LRS associated with SO_2_ exposure. Early identification and triaging of individuals possessing these risk factors in order to prioritise evacuation may be an important preventive public health measure. The study also demonstrated that BHR persists in a large proportion of affected individuals 6 years after the initial exposure to high levels of SO_2_, pointing to the importance of post-incident medical surveillance to identify affected individuals that may need further health care. It is recommended that future studies also consider additional parameters when modelling exposures so as to derive more reliable exposure estimates in order to explore dose-response relationships in a more meaningful manner, so as to identify threshold effects and other exposure reduction strategies in affected communities.

## Figures and Tables

**Table 1:** Demographic characteristics of study population exposed to the sulphur stockpile fire incident.

Demographic Characteristics	Case	Controls
	(*n* = 76)	(*n* = 180)
Age (yrs)	43 ± 12	41 ± 13
Gender		
Males	23 (30%)	63 (35%)
Females	53 (70%)	117 (65%)
Smoking status		
Non-smoker	33 (43.4%)	73 (40.5%)
Ex-smoker	29 (38.2%)	55 (30.5%)
Current smoker	14 (18.4%)	52 (29%)
Previous tuberculosis *	13 (17%)	10 (6%)

Note: Categorical variables–number (%), Continuous variable–mean ± S.D. * Reported pulmonary tuberculosis >1 year prior to the incident.

**Table 2:** Prevalence of obstructive lung disease among cases exposed to the sulphur stockpile fire incident.

Obstructive Lung Disease Based on Spirometry	Prevalence (%)
	(*n* = 76)
FEV_1_ increase post-bronchodilator (≥12% and ≥200 ml)	28 (37%)
FEV_1_/FVC ratio ≤70% *	18 (24%)
Impaired lung function: FEV_1_ <80% predicted *	48 (63%)
Mild: 50–79% predicted	42 (55%)
Moderate: 30–49% predicted	4 (5%)
Severe: <30% predicted	2 (3%)

Note: Categorical variables–number (%), Continuous variable–mean ± S.D. * Post bronchodilator ECCS (1993) prediction equations used for lung function reference values.

**Table 3:** Summary exposure metrics for sulphur dioxide exposures in the study population at the time of the sulphur stockpile fire incident.

Cases (*n* = 76)					Controls (*n* = 180)				
Exposure Metric	Mean ± S.D.	Range	Median	IQR	Mean ± S.D.	Range	Median	IQR	*p*-Value
Total hours of exposure	8.43 ± 6.33	1–21	6	6.5	8.54 ± 6.11	1–21	6	7	0.896
Peak exposure (ppm)	46.46 ± 86.67	0–444.06	6.94	64.51	40.63 ± 95.91	0–540.64	4.01	41.17	0.649
Cumulative exposure (ppm×hrs)	104.22 ± 168.46	0–821.97	13.37	163.78	101.40 ± 187.70	0–884.23	7.74	106.95	0.910
Outside dwelling at the time of the fire	26 (34%)				63 (35%)				0.904

*t*-test for continuous variables, chi-square test for categorical variables.

**Table 4:** Relationship between host factor attributes associated with persistent lower respiratory symptoms/asthma in subjects exposed to the sulphur stockpile fire incident.

Host Factors	Odds Ratio	95% CI	*p*-Value
Age	1.01	0.99–1.03	0.458
Gender			
- Female	1.24	0.70–2.21	0.464
Smoking status			
- Non-smokers	1.00		
- Ex-smokers	1.20	0.65–2.21	0.554
- Current smokers	0.61	0.30–1.26	0.186
Previous Pulmonary TB *	3.49	1.46–8.35	0.005
Self-reported acute symptoms:			
*Ocular-nasal symptoms*			
- burning eyes	0.77	0.45–1.33	0.345
- burning/sore nose	0.91	0.50–1.66	0.770
- burning/sore throat	0.93	0.49–1.79	0.836
*Lower respiratory symptoms*			
- burning/sore chest	1.47	0.79–2.74	0.218
- cough	1.17	0.65–2.09	0.602
- shortness of breath	1.97	0.95–4.09	0.070
- tight chest	9.93	5.15–19.11	<0.001
*Constitutional symptoms*			
- headache	0.27	0.08–0.92	0.036
*Gastro-intestinal symptoms*			
- nausea/vomiting	0.60	0.25–1.45	0.257
- diarrhoea	0.79	0.08–7.69	0.837

* Reported pulmonary tuberculosis >1 year prior to the incident.

**Table 5:** Relationship between environmental factors associated with persistent lower respiratory symptoms/asthma in subjects exposed to the sulphur stockpile fire incident.

Using Logistic Regression Models	O.R.	95% CI	*p*-Value
***Unadjusted models***			
**Location at time of incident**			
Outside v/s inside	0.97	0.55–1.70	0.904
**Total hours of exposure (hrs) ^a^**			
5–6 hrs	1.48	0.72–3.05	0.291
7–11 hrs	0.95	0.44–2.04	0.900
>11	1.06	0.50–2.26	0.880
**Cumulative exposure (ppm×hours) ^b^**			
6–50 ppm	0.79	0.39–1.61	0.509
>50 ppm	1.40	0.76–2.57	0.282
**Peak exposure (ppm) ^c^**			
2–20	1.02	0.50–2.01	0.964
>20	1.42	0.77–2.62	0.267
**Hourly exposures**			
Exposure at hour 7 (hr_7)_	1.00	0.99–1.00	0.695
Exposure at hour 15 (hr_15_)	1.03	0.99–1.06	0.060
Adjusted models *			
**Hourly exposures**			
Exposure at hour 7 (hr_7_)	1.00	0.99–1.00	0.338
Exposure at hour 15 (hr_15_)	1.04	1.01–1.07	0.021

Note: ^a^ baseline < 5 h of exposure, ^b^ baseline < 6 ppm, ^c^ baseline < 2 ppm. * Adjusted for age, gender, smoking and previous PTB > 1 year prior to the incident.
